# Identification of the genes at *S* and *Z* reveals the molecular basis and evolution of grass self-incompatibility

**DOI:** 10.3389/fpls.2022.1011299

**Published:** 2022-10-18

**Authors:** Rowan Herridge, Tyler McCourt, Jeanne M. E. Jacobs, Peter Mace, Lynette Brownfield, Richard Macknight

**Affiliations:** ^1^ Department of Biochemistry, University of Otago, Dunedin, New Zealand; ^2^ Forage Science, AgResearch, Christchurch, New Zealand

**Keywords:** grass, self-incompatibility, DUF247, pollen, reproduction, Poaceae, stigma

## Abstract

Self-incompatibility (SI) is a feature of many flowering plants, whereby self-pollen is recognized and rejected by the stigma. In grasses (Poaceae), the genes controlling this phenomenon have not been fully elucidated. Grasses have a unique two-locus system, in which two independent genetic loci (S and Z) control self-recognition. S and Z are thought to have arisen from an ancient duplication, common to all grasses. With new chromosome-scale genome data, we examined the genes present at S- and Z-loci, firstly in ryegrass (*Lolium perenne*), and subsequently in ~20 other grass species. We found that two DUF247 genes and a short unstructured protein (SP/ZP) were present at both S- and Z- in all SI species, while in self-compatible species these genes were often lost or mutated. Expression data suggested that DUF247 genes acted as the male components and SP/ZP were the female components. Consistent with their role in distinguishing self- from non-self, all genes were hypervariable, although key secondary structure features were conserved, including the predicted N-terminal cleavage site of SP/ZP. The evolutionary history of these genes was probed, revealing that specificity groups at the Z-locus arose before the advent of various grass subfamilies/species, while specificity groups at the S-locus arose after the split of Panicoideae, Chloridoideae, Oryzoideae and Pooideae. Finally, we propose a model explaining how the proteins encoded at the S and Z loci might function to specify self-incompatibility.

## Introduction

Self-incompatibility (SI) is a mechanism by which plants are able to distinguish, and reject fertilization of self pollen. SI is mediated by hypervariable genes, expressed in male tissue (anther/tapetum/pollen) and female tissue (pistil/stigma) which interact at the pollen/stigma interface. The genes underlying SI have been described in Solanaceae, Brassicaceae and Papaveraceae, revealing different mechanisms in these families (Foote et al., 1994; Schopfer et al., 1999; Kachroo et al., 2001; Kao and Tsukamoto, 2004). Despite the agronomic importance of the grass family (Poaceae), the genes underlying SI have not been elucidated. Self-fertility is an important domestication trait, and the ability to inbreed plants is highly desirable for F1 hybrid breeding (Glemin and Bataillon, 2009; Herridge et al., 2019).

Grasses have a unique SI system, which involves two genetic loci (*S* and *Z*), both of which must be matched in pollen/stigma in order to prevent self-fertilization; however, the underlying molecular mechanism remains unresolved. This system acts gametophytically, which means that the genotype of the haploid pollen contributes to SI response, and SI determinants are produced by the pollen grain itself. This is in contrast to the *Brassica* system, which acts sporophytically, and the SI determinants are deposited on the surface of the pollen grain by the diploid tapetum, preventing fertilization based on the genotype of the paternal (sporophytic) parent, regardless of which allele the pollen grain inherits (Hiscock and McInnis, 2003). The grass system also acts by a self-recognition system, whereby interaction of self pollen on the stigmatic exudate quickly leads to pollen tube arrest (Shivanna et al., 1982). This is in contrast to a non-self-recognition system, as is seen in the Solanaceae family, whereby non-self SI determinants (stigmatic S-RNase in the case of Solanaceae) are recognized by pollen-expressed F-box proteins leading to their degradation, while self S-RNase is not recognized and results in pollen tube arrest (Fujii et al., 2016). Evidence for this non-self mechanism comes from tetraploid grasses, in which SI is maintained, and requires only that one S- and one Z-locus is matched in the stigma (by contrast, tetraploid Solanaceae are self-compatible; Stout and Chandler, 1941; Fearon et al., 1984; Baumann, 2000). Therefore, a positive S/Z interaction is essential to grass SI, and cannot be complimented by a dual S (S/S) or Z (Z/Z) interaction. This suggests that the underlying genes have distinct functions in the downstream signaling pathway that must be integrated to result in pollen tube arrest. The two locus system is thought to have arisen from a duplication occurring in the common ancestor of grasses, although how a one-locus system would operate given the necessity of S/Z integration in modern grasses is an open question (Lundqvist, 1962).

Mapping of the S- and Z-loci in grasses has been performed in a number of species (Yang et al., 2008). In ryegrass (*Lolium perenne*), a DUF247 protein co-segregated with the S-locus, and had variable sequence between different specificity groups (plants which are capable of crossing with one another, but not themselves; Manzanares et al., 2016). Importantly, this gene was predicted to be non-functional in a self-compatible relative of ryegrass, *L. temulentum* (Manzanares et al., 2016). In addition, no other hypervariable genes were found in the predicted region based on the draft genome sequence developed by Byrne et al. (2015). Similarly, a DUF247 gene was located at the Z-locus in *L. perenne*, although variability of this gene was not probed (Shinozuka et al., 2010; Thorogood et al., 2017). In a SI rice species, *Oryza longistaminata*, two DUF247 genes, and a small pistil-expressed protein (named SP) was present at the S-locus (Lian et al., 2021). With the advent of a high-quality ryegrass genome, as well as abundant high-quality grass genomes now publicly available, we aimed to probe these regions in detail using prior knowledge from the above studies (Frei et al., 2021; Nagy et al., 2022).

Our work revealed that three genes are likely key to the function of both the S- and Z- loci; two DUF247 genes and a gene encoding a short unstructured protein (SP and ZP, respectively). We propose a model where a pollen extracellular receptor, comprising two S- and two Z- DUF247 proteins, specifically recognizes its own stigma excreted SP/ZP signal proteins, activating a pathway that prevents fertilization by its own pollen. SC grasses likely evolved through mutation or loss of these key SI components.

## Results

### Synteny between the S- and Z- loci indicate that DUF247 and SP/ZP genes are responsible for ryegrass self-incompatibility

**Figure 1:**
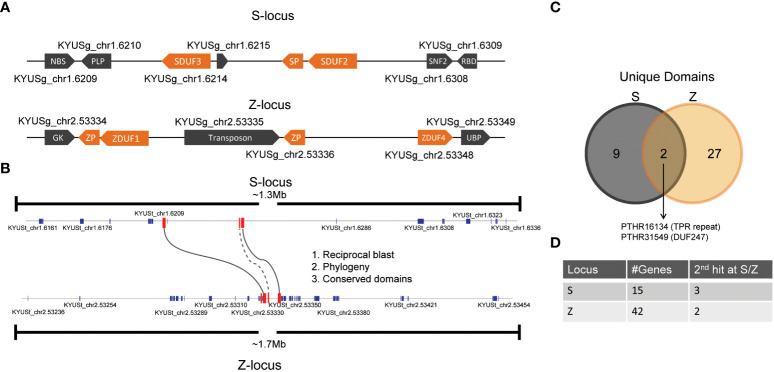
Synteny between S- and Z- loci in L. perenne. **(A)** Structure of the DUF247/SP/ZP at S- and Z-loci in the Kyuss genome (Frei et al., 2021). **(B)** Annotated genes in a 1.3 and 1.7 Mb region around SDUF247 and ZDUF247 were compared by phylogenetic analysis (see **Supplementary Figure 1**), BLAST searching and comparing domains in common. Only the DUF247 genes passed all three tests, indicated by solid lines. SP had a second tBLASTn hit at the Z-locus, indicated by a dotted line. **(C)** Number of unique domains identified at the S- and Z-loci and the overlap between the two. **(D)** Number of annotated genes in each region, and the number with secondary tBLASTn hits at the opposing (S/Z) locus.

With the recent publication of a high-quality ryegrass genome, we set out to investigate candidate genes that might control self-incompatibility (Frei et al., 2021). DUF247 genes have been identified at both the S- and Z-loci of ryegrass as likely candidates for mediators of SI (Manzanares et al., 2016; Thorogood et al., 2017). Ryegrass contains a large number of DUF247 genes, four DUF247 genes (DUF1-4) form a small clade including DUF2, the aforementioned DUF247 associated with the S-locus (Veeckman et al., 2019). Further supporting the role of the DUF247 genes, two DUF247 genes have been identified at the S-locus of the self-incompatible wild perennial rice species *O. longistaminata* (Lian et al., 2021). Alongside the DUF247 gene, a gene encoding a short protein dubbed *S-LOCUS PISTIL* (SP) was also identified as a potential candidate gene within the *O. longistaminata* S-locus, as the SP gene was highly polymorphic and expressed at high levels in the pistil tissue (Lian et al., 2021). As grass SI loci are predicted to have arisen from a duplication, we expected the nature of the genes present at the S- and Z-loci to be similar (Lundqvist, 1962). We examined the sequence at the S- and Z-loci in the recently published high-quality ryegrass genome, using tBLASTn with DUF247 proteins from *L. perenne* and SP proteins from *O. longistaminata*, revealing two DUF247 genes at each location (**Figure 1A**; Frei et al., 2021). The flanking genes of SDUF247 in the Kyuss genome were the same as those identified by Manzanares et al. (2016). The DUF247 genes at S- and Z- were the small clade mentioned above, described in Veeckman et al. (2019) and named DUF1-4. We retained this nomenclature, and labeled them with their corresponding locus, yielding SDUF2, SDUF3, ZDUF1 and ZDUF4. We also identified one or two SP-like genes at each location and retained this nomenclature, naming the relevant genes “SP” and “ZP” (**Figure 1A**). In cases where two ZP-like genes were identified, they were named ZP1 or ZP4 by their proximity to either ZDUF1 or ZDUF4.

The leading hypothesis for the origin of the S- and Z-loci in grasses is that they evolved from a precursor, single locus (Lundqvist, 1962); using this prediction, we aimed to rule out the possibility of other genes in the S- and Z-loci controlling SI specificity. To determine if DUF247/SP/ZP were the only genes in common between S- and Z- we took a region >1Mb centered around the S- and Z-DUF247 genes (on Chromosome 1 and 2; **Figure 1B**), and extracted all predicted proteins from the *L. perenne* (Kyuss) genome (Frei et al., 2021). Using Interpro to predict protein domains we compared proteins from S- and Z- to identify overlapping protein types. This revealed only DUF247 genes and an FBOX/TPR-repeat gene are present at both loci (**Figure 1C**; **Supplementary Dataset S1**). Aligning the two FBOX/TPR-repeat proteins did not show any regions of substantial alignment, and these genes were not linked in the other two tests (described below). As the SP/ZP proteins do not have predicted domains, they were not included in this analysis. To attempt to address this, and detect other proteins without annotated domains, we produced a phylogenetic tree from the entire list of proteins, with the anticipation that highly similar proteins from S- and Z- may cluster together, but with the caveat that highly polymorphic S/Z-determinants may not cluster or that the high divergence would confound the tree. Nevertheless, the phylogenetic tree produced a cluster with 4 DUF247 proteins showing the highest similarity of any cluster containing proteins from Chromosomes 1 and 2 (**Supplementary Figure S1**). SP/ZP did not cluster together in this analysis, likely owing to their short size and high degree of polymorphism. Finally, in the case that a protein may only be annotated at one locus, and not the other (as annotations are often produced bioinformatically, unusual genes are occasionally omitted), we performed tBLASTn using protein sequences from S- and Z- with the expectation that the top blast hit would be the native position of each protein, and the second hit would be at the most recent duplication – in the case of S- and Z-determinants this would likely be at the Z- or S- locus, respectively. As expected, the first hit of all predicted proteins was at their native location, and only in the case of the four DUF247 proteins and the SP protein was the second hit at the S- or Z-locus (**Figure 1D**; **Supplemental Dataset S2**). The fact that no other genes were in common between S- and Z-loci provides evidence that the four DUF247 genes and the SP/ZP genes were responsible for SI determination.

### The expression of the DUF247 and SP/ZP genes in reproductive tissues is consistent with the role in determining self-incompatibility

To mediate specificity, the DUF247 and SP/ZP genes must be expressed in the pollen or stigmatic tissues. Previous experiments from ryegrass indicated that SDUF2 is present in pollen, although with some stigmatic expression (Manzanares et al., 2016). Analysis of SDUF2 (named *OlSS2*) and SDUF3 (*OlSS1*) and SP from *O. longistaminata* suggested SDUF3 was pollen-specific, SDUF2 expressed in both pollen and pistil, and SP strongly pistil-specific (Lian et al., 2021). One limitation of the prior RNA-seq data analysis in ryegrass was the absence of an appropriate reference genome for identifying transcript abundance from highly variable genes. “SP” for example, was omitted from the analysis performed by Manzanares et al. (2016) likely owing to the inability to map it to the reference genome, despite its presence in the draft genome and RNA-seq data from this study (Byrne et al., 2015). We performed RNA-seq on developing anthers and stigma from a heterozygous *L. perenne* individual from the ONE50 cultivar with a draft genome assembly (referred to as “ONE50a” henceforth; J.M.E. Jacobs unpublished data). Firstly, we aimed to identify well-assembled S- and Z- alleles using BLAST with DUF1-4 as queries. Two S- and Z- alleles were found, as expected of a heterozygous individual, however, only one of each was well-assembled and selected for further analysis (on scaffold_1841 and scaffold_1554). We also screened other ONE50 plants in our collection for the presence of S- and Z-loci that matched this genome using locus-specific primers to the DUF/SP/ZP genes, identifying an individual matching the Z-locus on scaffold_1554.

**Figure 2:**
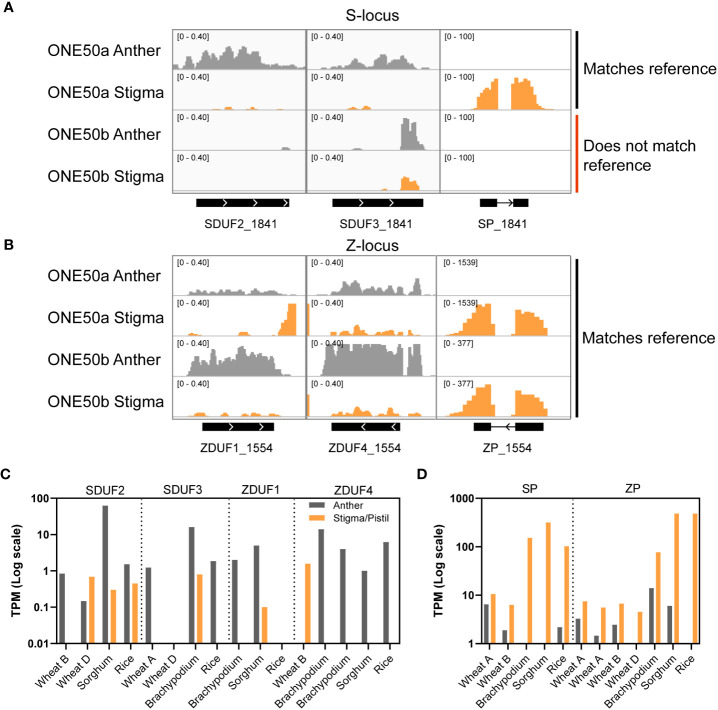
Expression of DUF247 and SP/ZP in reproductive tissues from grasses. **(A, B)** RNAseq coverage at the S- **(A)** and Z-locus **(B)** in anther and stigma tissue from two ONE50 individuals. One plant (ONE50a) was a clone of the individual used to create the reference sequence, and had the corresponding genotype at S- and Z-, while the other (ONE50b) only had the corresponding Z-locus genotype. Numbers in the top left of each sub-panel indicate the y-axis scale in units of coverage in reads per million over 25bp windows. **(C, D)** Expression of DUF247 **(C)** and SP/ZP **(D)** genes from rice, wheat, sorghum and brachypodium in anther and stigma (or pistil) from expression databases.

Consistent with prior results, SDUF2 was primarily anther-expressed, but with some stigmatic expression, while SDUF3 was more anther-biased (**Figure 2A**). SP was exceptionally highly expressed in stigma (**Figure 2A**). At the Z-locus ZDUF1 was anther-specific, while ZDUF4 was expressed in both tissues (**Figure 2B**). ZP was strongly expressed in stigma (**Figure 2B**). A second putative “ZP” locus was not expressed, suggesting that only one ZP is required for SI, and the presence of a second ZP may be a relic of the single-locus system. As mentioned above, only DUF/SP/ZP sequences that matched the reference ONE50 genome were able to be aligned, evidenced by the second sequenced plant (labeled “ONE50b”) only showing reads aligning to the Z-locus on scaffold_1554, and not the S-locus on scaffold_1841 (**Figure 2A and B**). To assay the abundance of unmapped DUF/SP/ZP transcripts in both individuals, we assembled transcripts *de novo* from this RNA seq data. In both plants (ONE50a and ONE50b), four SP/ZP and eight S/ZDUF transcripts were assembled; however, in two cases, the ZDUF transcript, and associated ZP transcript were assembled into a single contig (**Supplementary Figure 2**). In these instances, ZP expression was likely the predominant factor influencing the expression value (reflected in high stigma expression for these transcripts (**Supplementary Figures 2B, D**). Nevertheless, the expression pattern for these transcripts matched the genome-guided approach above, with DUF247 genes expressed in anthers, and SP/ZP highly expressed in stigma (**Supplementary Figure 2**). Combined, transcripts from SP and ZP made up a large proportion of the transcriptome in our datasets (~26,000 TPM and ~10,000 TPM in each stigma dataset).

To determine if the DUF247 gene is expressed in the male and SP/ZP genes are expressed in the female reproductive tissues in other grasses, we examined expression results using available data from other grass species. Anther and stigma/pistil transcriptomic data was accessible for *Brachypodium distachyon*, *O. sativa*, *Triticum aestivum* and *Sorghum bicolor* (Davidson et al., 2012; Ma et al., 2021). We identified SP/ZP and DUF247 genes from these species using tBLASTn querying the genomic sequence of each species. In all species, S/ZDUF247 and SP/ZP were identified in close proximity, similar to *L. perenne*, indicating that we had identified the correct loci. Some of the genes were annotated (**Supplementary Table 1**), allowing us to access the available transcriptomic data for these species. Expression patterns of these genes in all species were broadly consistent with those seen in *L. perenne* – modest anther-specific expression of DUF247 and high stigma/pistil expression of SP/ZP (**Figures 2C** and **D**). In summary, the expression data from ryegrass and a range of other grasses is consistent with the DUF247 genes function as the anther component and the SP/ZP genes function as the stigma component of the SI mechanism.

### Disruption of the DUF247 and SP/ZP genes in self-compatibility highlights their importance for SI

**Figure 3:**
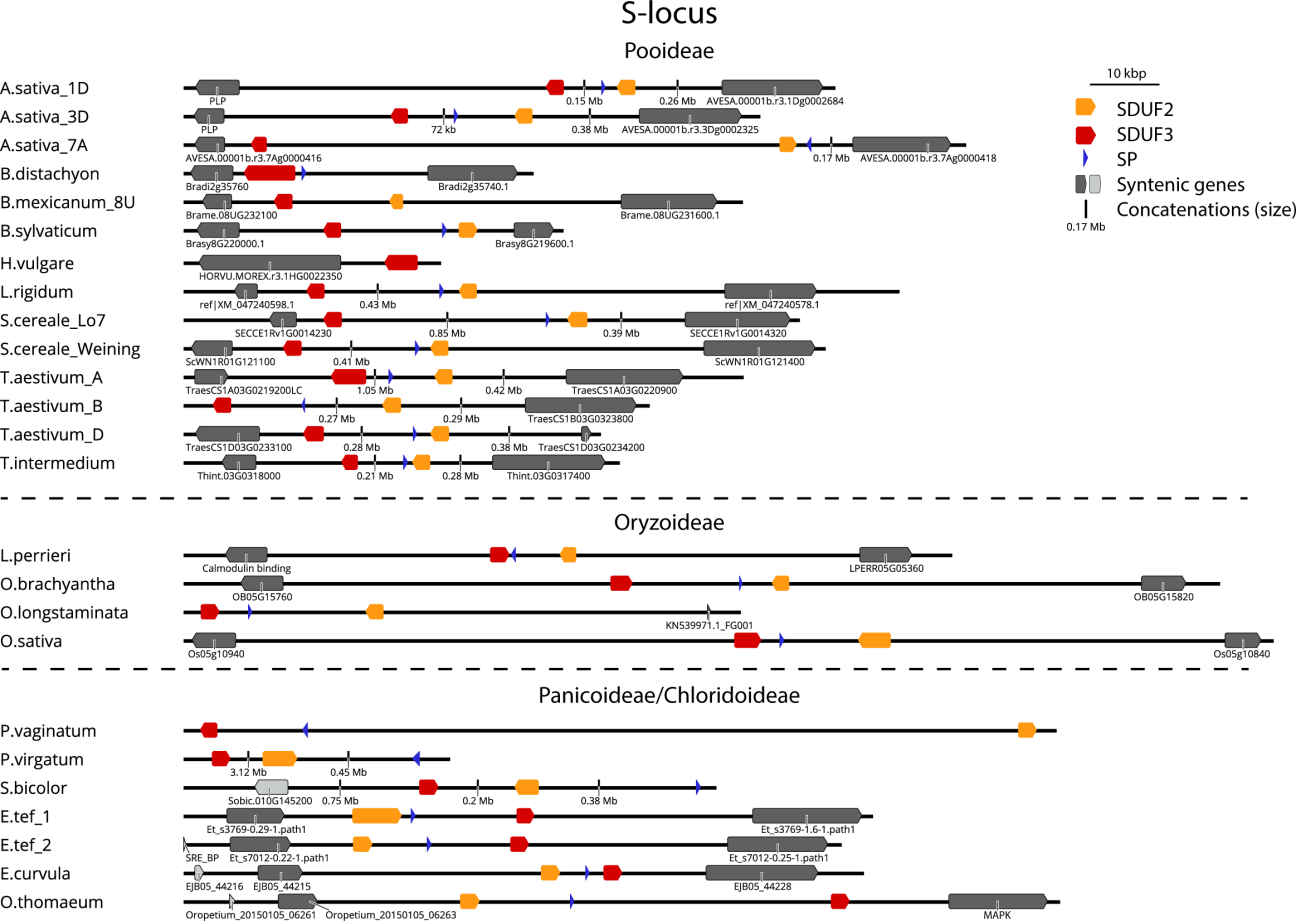
Structure of the S-locus in different grass subfamilies. Structure of S-loci from different species. Grasses of the Pooideae subfamily (top) are flanked by a Peridoxial phosphate homeostasis gene (PLP; left) and SWI/SNF chromatin remodeler (right). Oryzoideae grass S-loci are flanked by a gene encoding a calmodulin binding protein (left) and a zinc permease (right). Chloridoideae grasses (bottom) are flanked by a Sterol-response element binding protein (SRE-BP; left, light grey) and Flavin reductase (left, dark grey) and a Mitogen activated protein kinase (MAPK, right). Panicoideae grasses (*S. bicolor, P. virgatum, P. vaginatum*) did not show obvious synteny, except for SRE-BP in *S. bicolor*. Gene identifiers of flanking genes are shown where available. See **Supplementary Dataset S4** for details of genomes/versions.

**Figure 4:**
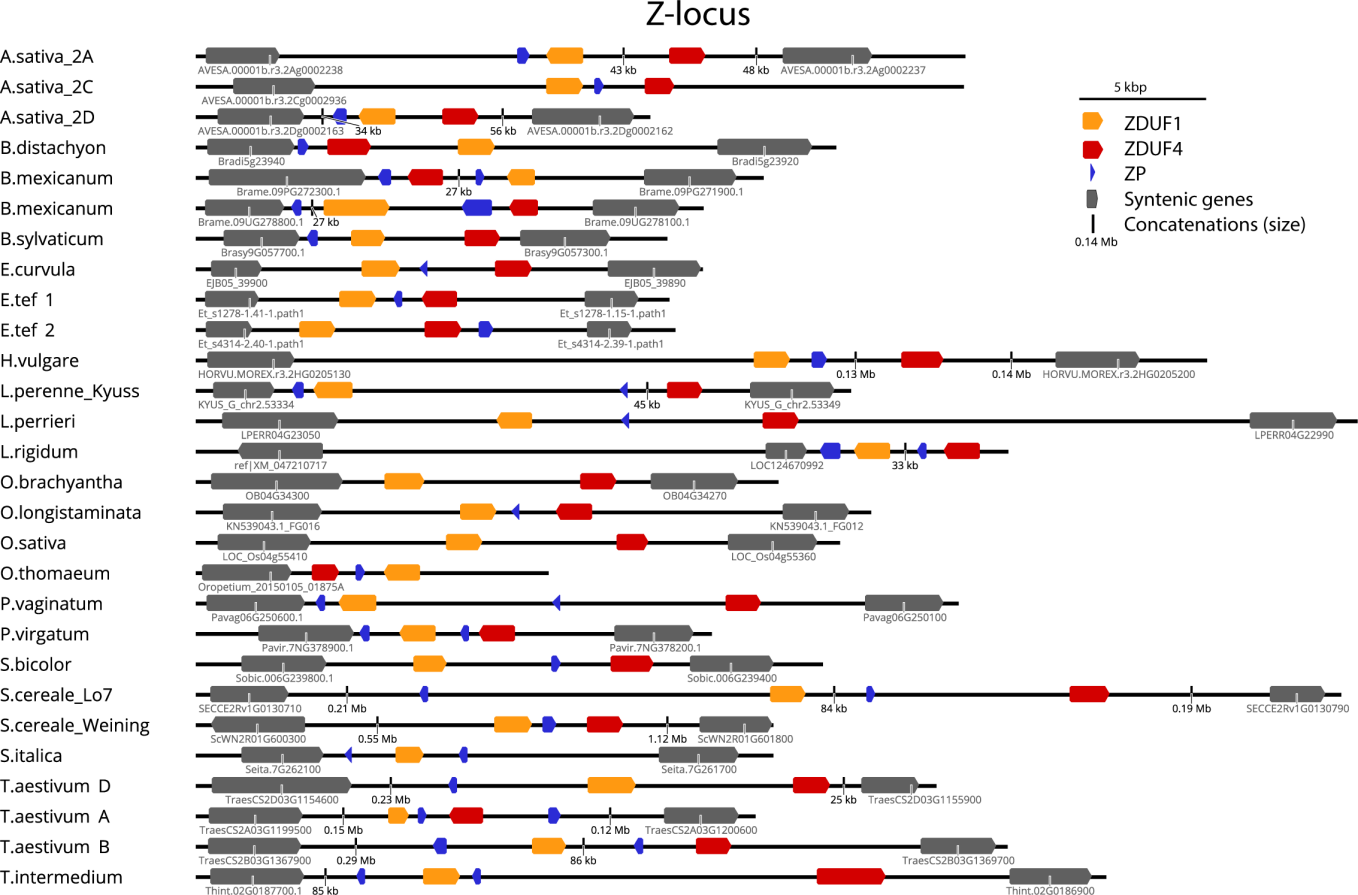
Structure of the Z-locus in different grass subfamilies. Structure of Z-loci identified in other species. In all cases, a Glycerol kinase (GK) is on the left, and Ubiquitin Conjugating Enzyme (UBC) is on the right. Gene identifiers are shown for GK and UBC for each genome where available. See **Supplementary Dataset S4** for details of genomes/versions.

Following the identification of DUF247/SP/ZP in the above species, we next searched available high-quality genomes for the syntenic regions from a large variety of SI and SC species. Should these genes be responsible for SI, they may be disrupted in SC species, either as the cause of SC, or alternatively through genetic drift upon loss of SI at loci outside S and Z. The genetic arrangement of two DUF247 and one or two SP/ZP genes was present in most species examined, allowing us to easily identify S- and Z-loci in these genomes (**Figures 3**, **4**). The flanking genes at the Z-locus were conserved in all species (A glycerol kinase (GK) and ubiquitin-conjugating enzyme (UBC); **Figure 1A**, **Figure 4**). The S-locus, however, appears to have different flanking genes in the Pooideae, Oryzoideae, Chloridoideae and Panicoideae, suggesting that it may have translocated in these clades, or alternatively, arisen independently (**Figure 3**).

Rearrangements were more common at the Z-locus than the S-locus, with the direction of DUF247 genes changing in relation to flanking genes. The size of the region also varied greatly, often correlating with genome size, from 16 kb in the *Oropetium thomaeum* Z-locus to >3 Mb at the *Panicum virgatum* S-locus (**Figures 3**, **4**). Importantly, all SI species examined contained functional DUF247 and SP/ZP at each locus, while all SC species had frame-shift mutations, or were missing elements (**Table 1**). One exception was the Weining *Secale cereale* genome, which contained functional versions of all genes – it is possible that a locus outside S- and Z- is responsible for SC in this genotype (Voylokov et al., 1998). In summary, disruption of the DUF247 and SP/ZP genes is a common mechanism for grasses to acquire self-compatibility and further supports the role of these genes in specifying SI.

**Table 1:** Presence of SI determinants in other grass species.

		SDUF2	SDUF3	SP	ZDUF1	ZDUF4	ZP^a^	Subfamily
	*Eragrostis curvula*	P	P	P	P	P	P	Chloridoideae
	*Hordeum bulbosum^b^*	P	P	P	P	P	P	Pooideae
	*Lolium perenne (Byrne)^c^*	P	P	P	P	P	P (x2)	Pooideae
	*Lolium perenne (Kyuss)*	P	P	P	P	P	P (x2)	Pooideae
	*Lolium perenne (Velmurugan)^d^*	P	P	P	P	P	A	Pooideae
	*Lolium rigidum*	P	P	P	P	P	P (x2)	Pooideae
	*Oryza longistaminata*	P	P	P	P	P	P	Oryzoideae
	*Panicum virgatum^d^*	P	P	P	P	P	P (x2)	Panicoideae
	*Paspalum vaginatum*	P	P	P	P	P	P (x2)	Panicoideae
	*Thinopyrum intermedium*	P	P	P	P	P	P (x2)	Pooideae
	*Avena sativa (A)*	P	P	P	P	P	P	Pooideae
	*Avena sativa (C)*	P	P	P	P	P	P	Pooideae
	*Avena sativa (D)*	P	P	P	P	P	P	Pooideae
	*Brachypodium distachyon*	A	P	P	P	P	P	Pooideae
	*Brachypodium mexicanum (P)*	NH	NH	NH	P	P	P (x2)	Pooideae
	*Brachypodium mexicanum (U)*	P	P	A	P	P	P	Pooideae
	*Brachypodium sylvaticum*	P	P	P	P	P	P	Pooideae
	*Eragrostis tef^e^*	P*	P	P	P*	P	P*	Chloridoideae
	*Hordeum vulgare*	A	P	A	P	P	P	Pooideae
	*Leersia perrieri*	P	P	P	P	P	P	Oryzoideae
	*Oropetium thomaeum*	P	P	P	P	P	P	Chloridoideae
	*Oryza brachyantha*	P	P	P	P	P	P	Oryzoideae
	*Oryza sativa*	P	P	P	P	P	P	Oryzoideae
	*Secale cereale (Lo7)*	P	P	P	P	P	P (x2)	Pooideae
	*Secale cereale (Weining)^f^*	P	P	P	P	P	P (x2)	Pooideae
	*Setaria viridis*	NH	NH	NH	P	A	P (x2)	Panicoideae
	*Sorghum bicolor*	P	P	P	P	P	P	Panicoideae
	*Triticum aestivum (A)*	P	P	P	P	P	P (x2)	Pooideae
	*Triticum aestivum (B)*	P	P	P	P	P	P (x2)	Pooideae
	*Triticum aestivum (D)*	P	P	P	P	P	P	Pooideae

P, present; Red shading indicates disruption of ORF, green shading indicates no disruption. A, absent. NH, no hits (entire locus was not identified).

a Occasionally more than one ZP gene was identified, marked by (x2).

b The Hordeum bulbosum genome is fragmented, no disruptions to DUF ORFs were found despite this.

c The sequenced plant was self-fertile, the location of the self-fertility allele is not published

d The Lolium perenne (Velmurugan) Z-locus was split across multiple scaffolds with ambiguous stretches contained within – the absence of ZP in this genome may be due to incompleteness

e Eragrostis tef is tetraploid, cases where one allele was interrupted are indicated by a *.

f Weining rye may contain SC mutations outside the S- and Z-loci.

### Protein structure predictions suggest that SI involves DUF247 proteins on the pollen tube membrane interacting with SP/ZP proteins secreted by the stigma.

We predicted that these genes would be hypervariable if they were specificity determinants, and that at least one component would be extracellular. Previous studies have shown variability in SDUF2, SDUF3 and SP (Manzanares et al., 2016; Lian et al., 2021). Indeed, alignments of DUF247 from available genomes (reconstructed in cases of frame shifting) showed hypervariability in two segments of each DUF247 member with limited variation elsewhere (**Supplementary Figure 3**). We used AlphaFold (Deepmind) software to produce a structure of *L. perenne* SDUF2, and subsequently mapped the variability of *L. perenne* SDUF2 sequence onto these structures (Veeckman et al., 2019; Mirdita et al., 2022). The two hypervariable regions of SDUF2 formed relatively unstructured loops (**Supplementary Figure 4**). These hypervariable unstructured loops may be involved in SP recognition, and/or oligomerization depending on the presence of other components of the SI system (e.g., SP or other DUF247 proteins).

SP and ZP were almost entirely hypervariable except for a few key residues including a C-terminal Cysteine, and a second cysteine 4-10AA away from the C-terminal (**Supplementary Figure 5**). Proline was common towards the C-terminal, as well as a conserved “EEK” motif in ZP proteins (**Supplementary Figure 5**). Small cysteine-rich peptides are common in SI signaling in Papaver and Brassica, likely due to their diffusible nature and ability to be transmitted easily through cell walls (Walker et al., 1996; Schopfer et al., 1999).While the primary sequences of these proteins were highly variable, some key features of secondary structure and functionality were conserved in both DUF247 and SP/ZP proteins, respectively. In the case of DUF247, the transmembrane domain at the C-terminal was well-conserved, suggesting that membrane localization is an essential component of its function, as previously proposed (Manzanares et al., 2016). In the case of SP/ZP, secondary structure prediction suggested that the N-termini typically contain elements of secondary structure (usually an alpha helix, although occasionally beta-sheet for a subset of ZP proteins; **Supplementary Figures 6A, B**). Signal peptide prediction software indicated that all SP and ZP were cleaved at the N-terminus, resulting in a short peptide (~65AA) which was expected to be extracellular (**Supplementary Figures 5C–F**; **Supplementary Dataset S3**). The only exception was ZP proteins from the PACMAD clade, where the predicted cleavage site did not pass statistical significance (**Supplementary Dataset S3**). While the cleavage site for ZP proteins occurred slightly closer to the N terminal, the resulting cleaved peptide was of similar length to SP cleaved peptides (**Supplementary Figuress 6D, E**). No GPI anchor was predicted in SP/ZP suggesting that these proteins may be free in the stigmatic exudate (**Supplementary Dataset S3**). Taken together, these results suggest that DUF247 proteins on the extracellular surface of the pollen tube membrane could interact with extracellular SP/ZP secreted by the stigma.

### Evidence for the co-inheritance and evolution of self-incompatibility components

**Figure 5:**
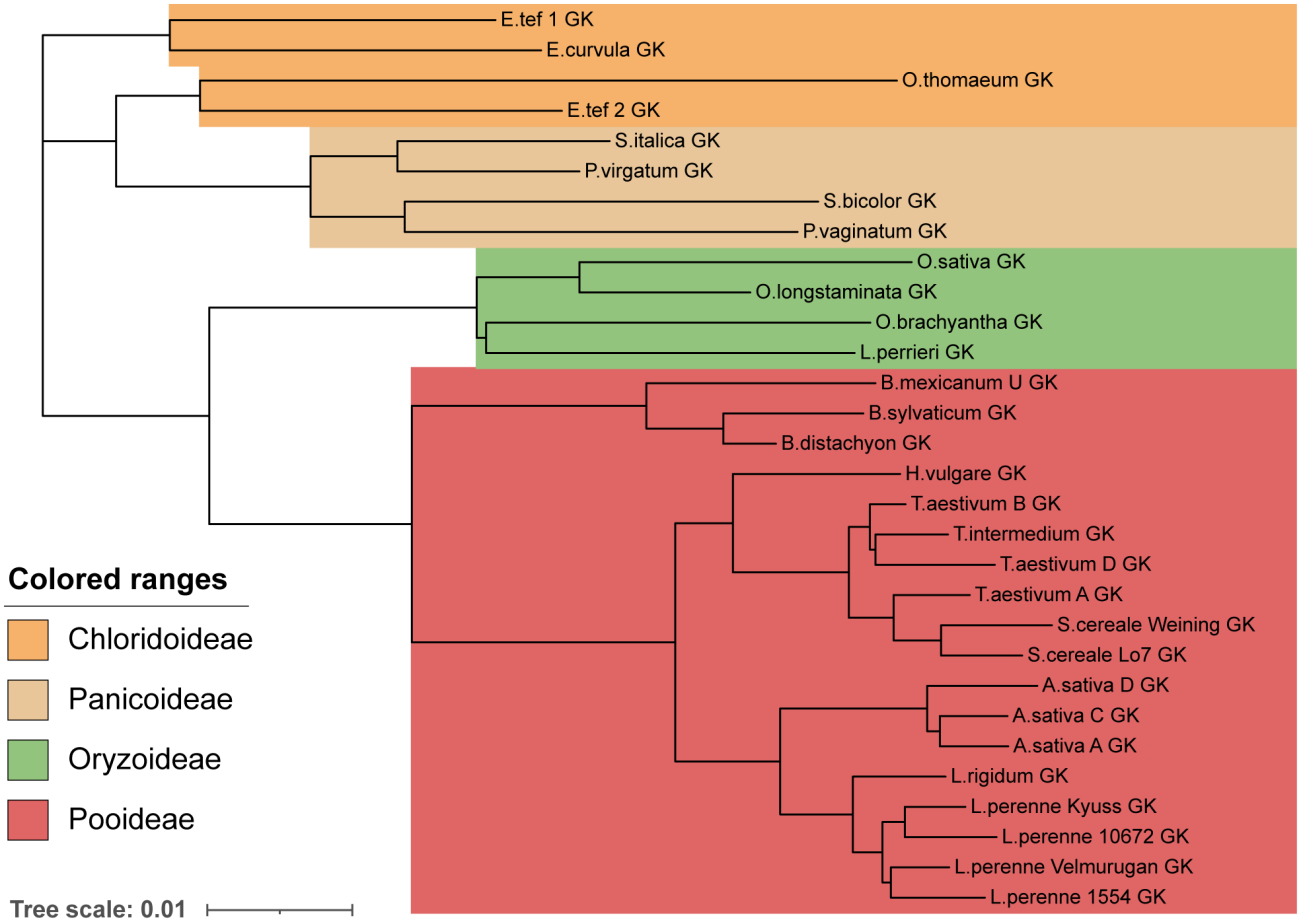
Phylogeny of grass species represented by Glycerol Kinase protein similarity. A phylogenetic tree constructed using the protein sequence of a Glycerol Kinase adjacent to all Z-loci. To produce the tree a global alignment with a BLOSUM62 cost matrix was performed, followed by tree building using a Jukes Cantor genetic distance model and nearest neighbor tree-building method. The phylogenetic tree resembles the expected relationship between species. Scale bar indicates substitutions per site.

Next, we aimed to examine the evolutionary history of these proteins. Two valid hypotheses that we aimed to probe were that the SI system and specificity groups originated before the divergence of a species, or, alternatively, that SI groups formed after the divergence of a species, meaning that each species would have a unique complement of DUF247 and SP/ZP alleles. As a control, we first constructed phylogenetic trees of the Glycerol Kinase genes adjacent to the Z-locus, as we predicted that these would resemble the known phylogeny of each species. This phylogenetic tree formed the expected clades of each family, including Panicoideae, Oryzoideae, Chloridoideae and Pooideae (**Figure 5**). In addition, closely related species within these families grouped together, such as the three wheat genomes (*T. aestivum*) with rye (*S. cereale*) and the three oat genomes (*Avena sativa*). If specificity groups arose before speciation, we expected different DUF/SP/ZP genes from various species to cluster together, regardless of the relatedness of the species. In contrast, if speciation occurred first, and specificity groups arose later, DUF247/SP/ZP from each species would clade together (in a manner similar to the GK phylogeny).

**Figure 6:**
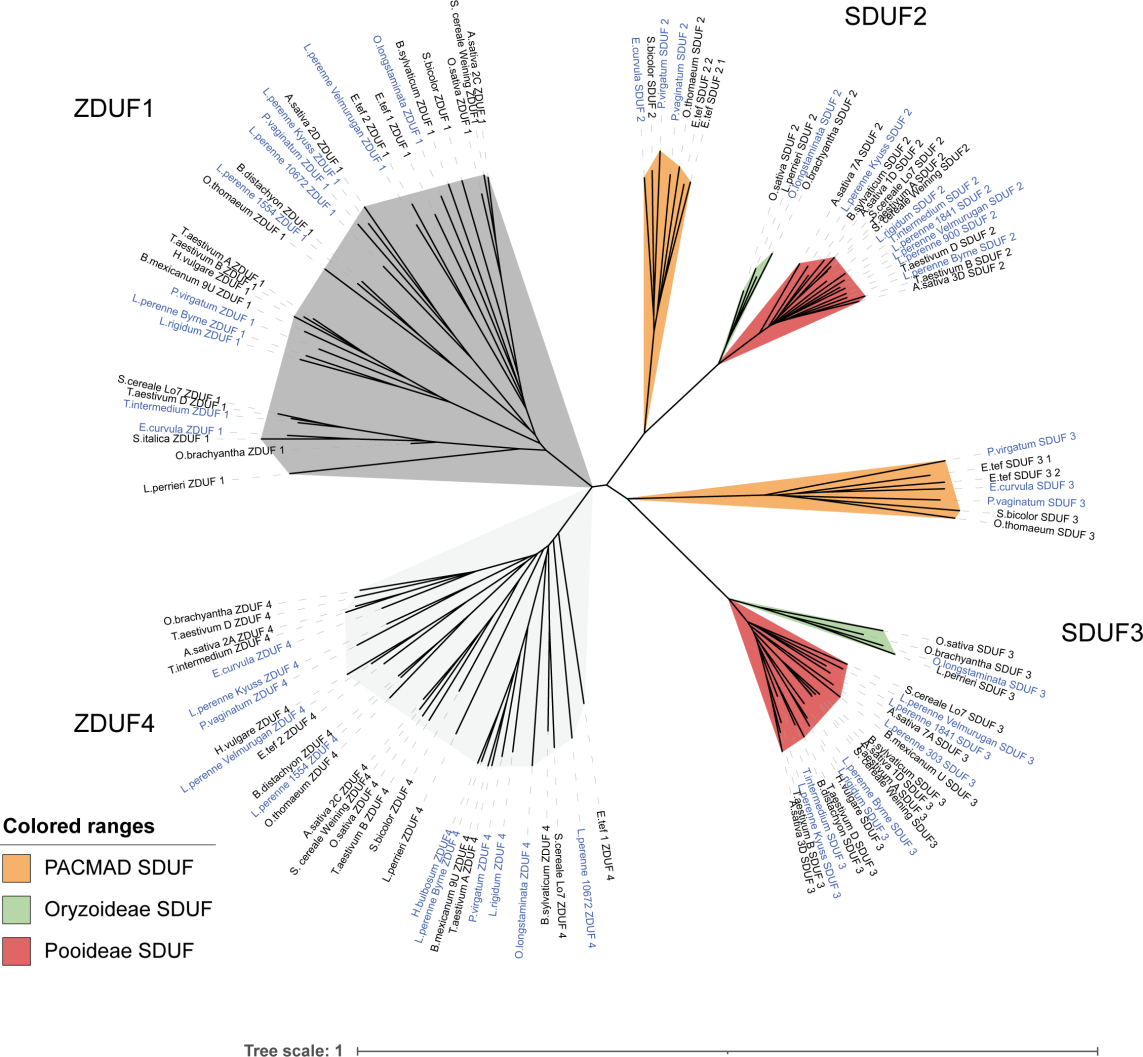
Phylogeny of S/Z-DUF247 genes from various grass species. A phylogenetic tree constructed using the sequences of all S/Z-DUF247 protein sequences. To produce the tree a global alignment with a BLOSUM62 cost matrix was performed, followed by tree building using a Jukes Cantor genetic distance model and nearest neighbor tree-building method. Subclades of SDUF2 and SDUF3 based on subfamily are shown. ZDUF1/ZDUF4 did not produce subclades. Self-incompatible species are highlighted with blue text. Scale bar indicates substitutions per site.

**Figure 7:**
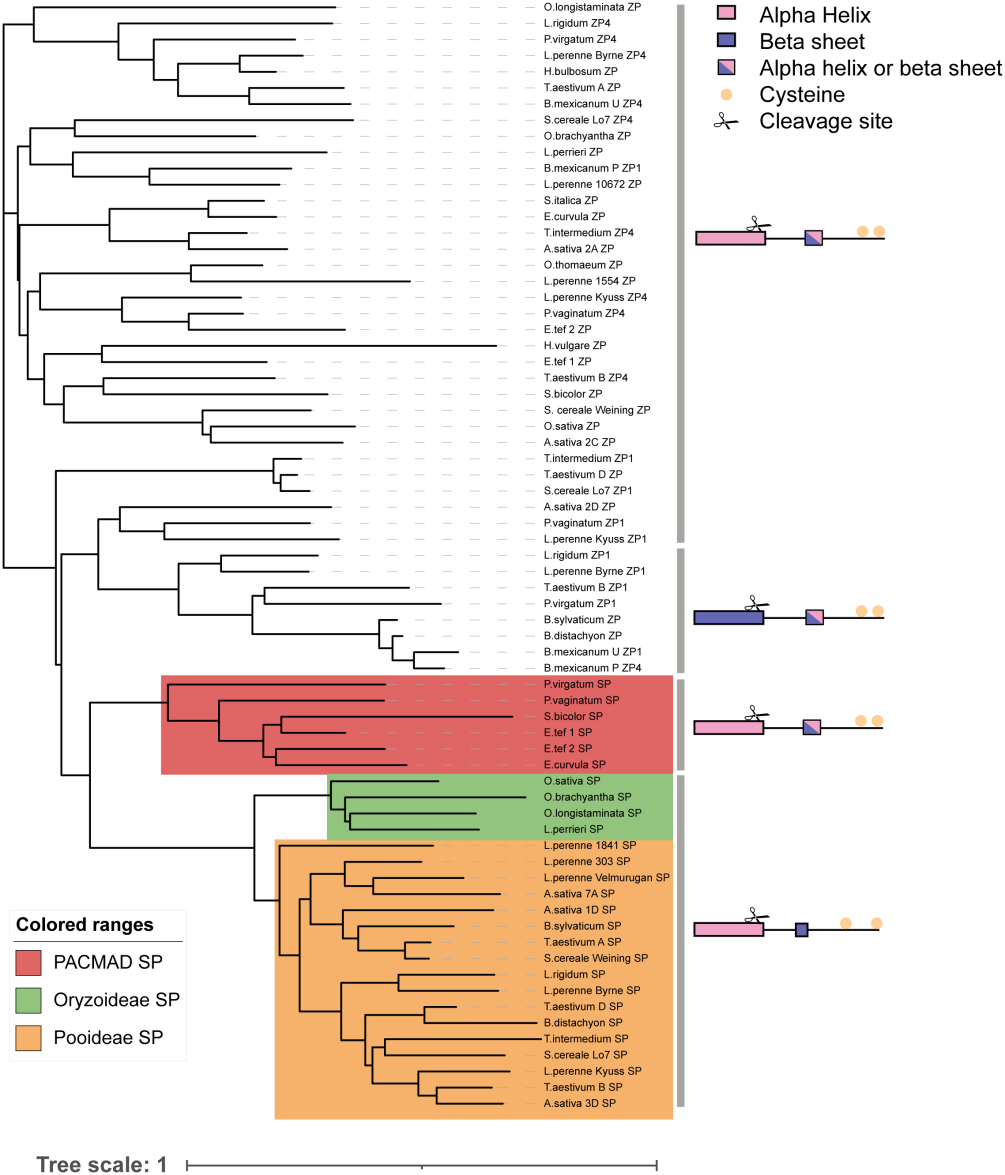
Phylogeny of SP/ZP genes from various grass species. A phylogenetic tree constructed using the sequences of all SP/ZP protein sequences. To produce the tree a global alignment with a BLOSUM45 cost matrix was performed, followed by tree building using a Jukes Cantor genetic distance model and nearest neighbor tree-building method. Representative structures, cleavage and cysteine location of each sub-clade shown at right. Scale bar indicates substitutions per site.

Firstly, we produced a large phylogenetic tree including all S/Z-DUF247 proteins, illustrating that the four key proteins are distinct between each grass subfamily (**Figure 6**). Given that all DUF247 proteins fell within one of the four (DUF1-4) clades, this indicates that the S- and Z-loci were inherited from a common ancestor, and that each locus (S- or Z-) has only originated once (**Figure 6**). In the case of the S-locus, however, specificity groups appear to have arisen after the divergence of Pooideae, Oryzoideae and the PACMAD clade (Chloridoideae and Panicoideae), as these clades are present in the SDUF phylogeny (**Figure 6**). However, specificity groups within the Pooideae (and likely within other clades) arose in a common ancestor and were subsequently inherited into all pooid grasses (versions of *T. aestivum* SDUF3 from the A, B and D genomes, for example, do not cluster together; **Figure 6**; and see **Supplementary Figures 7, 8**). These features were particularly evident when comparing multiple ryegrass SDUF2 proteins identified by Veeckman et al. (2019), with our collection of SDUF2 proteins from other species (**Supplementary Figure 7**). Despite the wide range of SDUF2 alleles from ryegrass, clades from Oryzoideae, Chloridoideae and Panicoideae were present; however, consistent with the idea that specificity groups arose in a common pooid ancestor, most pooid SDUF2 proteins had orthologs in the ryegrass panel (**Supplementary Figure 7**). Strengthening this argument, is the lack of synteny between Oryzoideae, Panicoideae and Pooideae S-loci (**Figure 3**). This is also reflected in the phylogeny of SP, where distinct clades for each subfamily are present (**Figure 7**).

At the Z-locus, however, speciation has occurred after specificity arose, evidenced by the mixing of different species throughout the tree (*S. bicolor*, from Panicoideae, for example, clusters with members of the pooid grasses, while *P. virgatum*, another Panicoideae, clusters elsewhere; **Figure 6**; and see **Supplementary Figure 9**). This was also reflected in the phylogeny of ZP, where mixtures of subfamilies cluster together (**Figure 7**). This is strengthened by the fact that all Z-loci examined were syntenic (occurring between UBC and GK proteins; **Figure 4**). Interestingly, sub-clusters of ZP including “ZP1” and “ZP4” also appear (so named by their proximity to ZDUF1 and ZDUF4), suggesting that ZP1 and ZP4 may be genuinely different proteins, perhaps interacting with their corresponding ZDUF protein (**Figure 7**).

From an evolutionary standpoint, the co-inheritance of SP/ZP and each DUF247 gene is essential to maintain SI. We compared SP and ZP phylogenetic trees with their corresponding S/Z-DUF247 trees to investigate whether these proteins were co-inherited, with the caveat that in SC species, these genes may have become unlinked. A strong association between SP proteins and SDUF3 was observed, but not so between SDUF2 and SP (**Supplementary Figure 8**). Smaller clades of linkage were observed for various ZP and ZDUF1/4 groupings (**Supplementary Figure 9**). Of particular note was the strong association between ZDUF4 and a small ZP4 clade, while ZDUF1 appeared to clade more similarly to ZP1 proteins (**Supplementary Figure 9**). This analysis is confounded by the fact that most species examined have potentially been SC for long periods and recombination may have occurred between, for example, SDUF2 and SDUF3/SP in many species. There is also uncertainty around the original/functional protein sequences in these species which have likely degraded over time, therefore impacting the creation of accurate phylogenetic trees. Nevertheless, the associations observed supports the idea that these genes, which are nearby, are co-inherited and evolve together.

## Discussion

**Figure 8:**
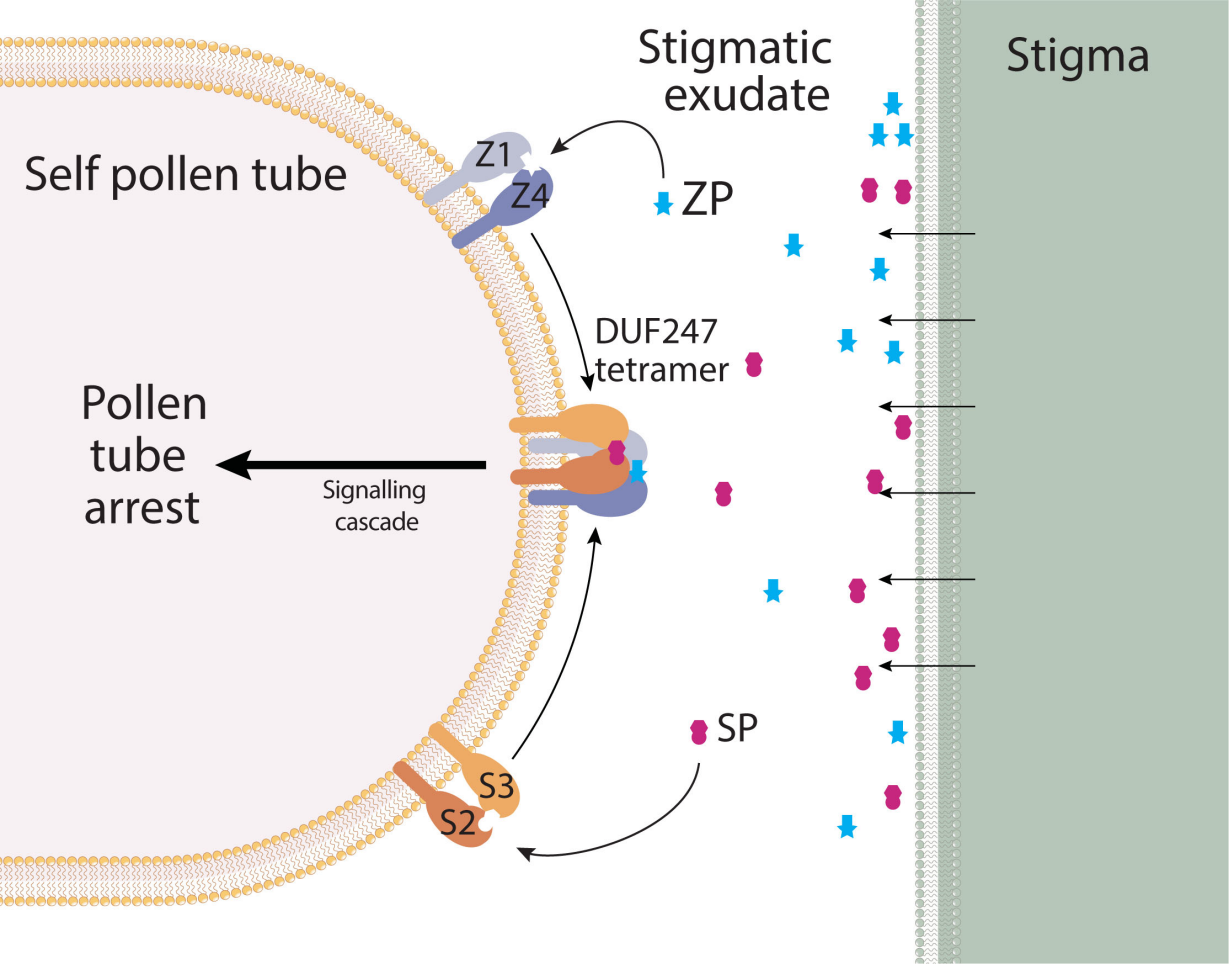
Model for SI in grasses. DUF247 proteins from S- and Z-loci form dimers at the pollen tube surface, and are anchored to the membrane by a conserved transmembrane domain. SP/ZP peptides are free-floating in the stigmatic exudate, allowing pollen tube arrest to occur immediately upon pollen germination. Signals from S- and Z- must be incorporated, therefore we propose a tetramerization of DUF247 proteins, resulting in a downstream signaling cascade that results in pollen tube arrest. Absence of any component, or non-self components would result in a lack of signaling, thus allowing pollen tube growth.

Here we describe the genes underlying the S- and Z-locus in Poaceae. The nature of each locus is similar, comprising two DUF247 genes, and a small gene encoding a cleaved, extracellular peptide. From this we propose a model which incorporates all six genes to instigate a self-incompatibility response in pollen (**Figure 8**). As both S- and Z-loci must match, we propose that a tetramer of DUF247 proteins forms upon recognition of self SP/ZP by DUF247 dimers made up of SDUF2/SDUF3 and ZDUF1/ZDUF4. This satisfies the requirement for the integration of S- and Z-signals leading to pollen-tube arrest. In *Brassica*, recognition of SCR/SP11 (a small cysteine-rich peptide) by SRK induces dimerization of SRK, forming a 2:2 SCR/SRK heterotetramer, providing a basis for our model (Ma et al., 2016). In addition, it is known that the grass SI response occurs very quickly after pollen tube germination. It is not required that the pollen tube reaches the stigmatic surface, or further down the style, providing support for SP/ZP being free-floating in the stigmatic exudate (Shivanna et al., 1982). Downstream integration of the S- and Z- signals may be occurring through known loci, for example the “SF locus” on chromosome 5 present in ryegrass and rye (Voylokov et al., 1998; Do Canto et al., 2018).

Evolutionary analysis of the DUF247/SP/ZP proteins suggested that the Z-locus was the original locus and S has resulted from a subsequent duplication (**Figure 6**). While the Z-locus appears to have retained a stable position, the S-locus has translocated and diversified between grass subfamilies (**Figure 3**). The presence of two ZP-like genes at some Z-loci suggests that a single locus system may comprise a “Z-locus” with two DUF247 genes and two ZP genes. Such a locus would have to have been capable of inducing pollen-tube arrest by itself, perhaps by forming a tetramer similar to our proposed S/Z-tetramer (**Figure 8**). Upon the advent of the S-locus, the second “ZP” gene has likely become redundant. Evidence for this appears in our data as a second ZP-like gene was present on scaffold_1554; however, it was not expressed in stigma or pollen samples. As the S-locus appears to have duplicated only once in the ancestral grass species, but diversified much later, it suggests that it may have had some function during this period. Modifiers are common in other SI species such as *SLG* in *Brassica* or *Sli* in *Solanum chacoense*, and these genes are often related or linked to the specificity determinants (Boyes et al., 1991; Eggers et al., 2021). One hypothesis is that the S-locus initially acted as an enhancer of SI response, until it later diversified, becoming a second determinant of specificity. Such an evolutionary pathway would satisfy the ability of the Z-locus to function independently prior to the duplication that resulted in the S-locus. Over time, presence of the S-locus could have allowed the Z-locus to lose its ability to signal pollen-tube arrest independently. One idea on the evolution of SI, is that this pathway shares a common ancestor with plant defence systems (Hiscock et al., 1996; Nasrallah, 2005). Intriguingly, overexpression of a DUF247 gene in Arabidopsis leads to SAR-like defense response, supporting this hypothesis for grass SI (Kondou et al., 2013).

Surprisingly, there is scant evidence for a two-locus system operating in the PACMAD clade. To our knowledge only *Miscanthus sinensis* has been analyzed for this purpose, showing a two locus system operates in this species (Jiang et al., 2017). Confounding this analysis is the extensive genome duplication in *M. sinensis*, and the apparent presence of two genomic loci that are similar to the Z-locus from other grasses on chromosome 11 and 12, with a putative SDUF gene on chromosome 18. None of the DUF genes in *M. sinensis* had full ORFs of the same size as other species – it may be that splicing is occurring in these genes, or alternatively, shortened DUF proteins may still be functional in *M. sinensis*. Until the S/Z-loci from *M. sinensis* have been mapped, this remains speculative. The large size of the “S-locus” in *S. bicolor* and particularly *P. virgatum* is also intriguing (**Figure 3**). In *P. virgatum* the SDUF3 gene is over 3Mb away from SDUF2, but SP is found within 500kbp of SDUF2, for example. Repression of recombination across large distances (>6 Mb) is also possible in plants, as evidenced by sex-loci in various species, which may also be occurring in the Panicoideae (Rifkin et al., 2021). The S-locus was much smaller in the three Chloridoideae species examined here (7kb in *Eragrostis curvula*, ~15kb in *E. tef* and 35kb in *O. thomaeum*). The functional *P. virgatum* Z-locus was present on Chromosome 7N, interestingly the version on Chromosome 7K contained many mutations, suggesting that upon becoming polyploid the second Z-locus has become non-functional. Similarly, *Paspalum vaginatum* had a second version of the S-locus on Chromosome 9 which contained many non-functional mutations, while the version on Chromosome 7 was fully intact (**Table 1**).

Our model, shown in **Figure 8**, proposes that SI involves all four DUF247 and at least one SP/ZP, with the hypervariability of each gene/protein determining the specificity of the SI mechanism. Further work is required to prove this model, such as showing that the DUF247 proteins interact physically to form S/Z tetramer and this complex recognizes self SP/ZP protein. We are currently undertaking these studies as well as using gene editing to determine if each of the S- and Z- components are essential for SI.

## Materials and methods

### Plant growth conditions

Ryegrass plants of ONE50 cultivar were grown at 22°C in 16h/8h light/dark cycles. To induce flowering, plants were placed at 4°C for 6 weeks with 8h/16h light/dark cycles. Plant ONE50a was a clonal copy of the individual plant used for generating all sequence data to generate the draft genome assembly of ONE50.

### Tissue collection and RNA preparation

Developing anthers were dissected from young flowers prior to dehiscence and placed on dry ice. Stigma were removed from flowers upon emergence and separated from ovaries and placed on dry ice before storing at -80°C. RNA was prepared using ~50mg of tissue. Frozen tissue was disrupted by bead bashing in a 1.5 mL Eppendorf tube with a stainless steel bead for 1 minute at 30 rps. Trizol (600 µL) was added to disrupted tissue, making sure to mix thoroughly as tissue thawed. The mixture was then centrifuged at 13,000 rpm for 1 minute, prior to further homogenization with a plastic micropestle. The fully-homogenized sample was centrifuged for a further minute, and the supernatant was transferred to a new tube with an equal volume of ethanol. RNA was extracted using a Zymo Quickzol miniprep kit according to manufacturer’s instructions, including on column DNAse treatment. RNA was eluted in 25 µL RNAse-free H_2_O.

### RNA sequencing

Sequencing and cDNA library preparation was performed by the Otago Genomics Facility. RNA was quality assessed using a bioanalyzer having a RNA integrity number >7. ~800 ng RNA was used to make cDNA libraries using the Illumina Truseq stranded mRNA kit. Libraries were sequenced using a NextSeq 2000 with 2x100bp paired-end read length.

### Mapping and analysis of RNA seq data

Fastq files were mapped to a draft ONE50 genome (J.M.E. Jacobs unpublished data) using STAR aligner v2.1.10a (Dobin et al., 2013). Alignment files (.bam) were processed in the Integrated Genomics Viewer (IGV) to create normalized count files with a window size of 25bp. Regions containing S- and Z-genes were identified by tBLASTn with DUF1-4 protein sequences as queries, followed by manual annotation of the SP/ZP genes (Veeckman et al., 2019). For *de novo* transcript assembly and quantification, Trinity software (v2.12.0) was used on combined anther and stigma datasets from each individual (“ONE50a” and “ONE50b”) under default settings with adapter trimming (Grabherr et al., 2011). Transcripts were then quantified using trimmed sequences from either anther or stigma using the salmon alignment-free estimation method (Patro et al., 2017).

### Identification of S- and Z-loci in other species

tBLASTn using DUF1-4 protein sequences was used to probe genome assemblies from various grass species (see **Supplementary Dataset S4** for details). The combination of DUF1/4 flanked by UBC and GK genes was used to classify a region as a Z-locus, while the combination of DUF2/3 flanked by SWI/SNF and PLP genes (Pooideae), calmodulin binding protein and zinc permease (Oryzoideae), or MAPK and Flavin reductase (Chloridoideae). Except where noted, genomes were accessed using Phytozome (https://phytozome-next.jgi.doe.gov/), GrainGenes (https://wheat.pw.usda.gov/), or Ensembl Plants (http://plants.ensembl.org/index.html). Ryegrass genomes were downloaded from online repositories and analyzed in the same manner with local copies (Byrne et al., 2015; Velmurugan et al., 2016; Frei et al., 2021). The following genome assemblies have been described in the primary literature *Eragrostis curvula* (Carballo et al., 2019), *E. tef* (Cannarozzi et al., 2014), *Hordeum vulgare* (Mascher et al., 2021), *Leersia perrieri*, *O. longistaminata, O.brachyantha* (Stein et al., 2018), *O. sativa* (Kawahara et al., 2013), *Oropetium thomaeum* (VanBuren et al., 2015), *Panicum virgatum* (Lovell et al., 2021), *Sorghum bicolor* (McCormick et al., 2017), *Secale cereale* (Lo7) (Rabanus-Wallace et al., 2021), *Secale cereale* (Weining) (Li et al., 2021), *Setaria italica* (Bennetzen et al., 2012), *Triticum aestivum* (Zhu et al., 2021).

### Secondary structure and feature prediction of SP/ZP proteins

Sequences of SP/ZP were aligned using ClustalW and secondary structure was analyzed using Ali2d software (Gabler et al., 2020). Signal peptide prediction was performed using SignalP 6.0 (Teufel et al., 2022). GPI anchor prediction was performed using NetGPI and subcellular localization prediction was performed using DeepLoc (Gíslason et al., 2021; Thumuluri et al., 2022).

### Protein prediction and phylogeny

Genes encoding DUF247 and SP/ZP were identified using tBLASTn on relevant genomes (**Supplementary Dataset S4**). In cases where DUF247 ORFs were disrupted, predicted sequences were reconstituted by aligning translated nucleotide sequences to the appropriate (functional) DUF247 protein, and subsequently concatenating amino acid sequences from each frame that aligned well. SP/ZP proteins were annotated by alignment to closely related SP/ZP sequences, detecting likely splice sites, and producing an in-frame SP/ZP protein, followed by recursively testing the proteins by alignment to other SP/ZP predicted proteins. Phylogenetic trees were created in Geneious (Biomatters) using parameters described in the legend of relevant figures. Trees were visualized in the interactive tree of life (iTOL; Letunic and Bork, 2021). Structural models were generated using the ColabFold implementation of AlphaFold (Jumper et al., 2021; Mirdita et al., 2022) and models were compared using ChimeraX (Pettersen et al., 2021).

## Data availability statement

RNAseq datasets generated in this study have been deposited to the NCBI Sequence Read Archive (SRA) BioProject ID PRJNA864892. Access to the ONE50 genome was provided by J.M.E. Jacobs (AgResearch, Lincoln) and may be available upon request.

## Author contributions

RH, RM, PM, and LB designed experiments and supervised the project. RH and TM carried out experiments. JJ provided the ONE50 draft genome assembly and a clonal copy of the plant used data to generate the draft genome assembly of ONE50. RH wrote the manuscript and produced figures. RM, PM, and JJ edited the manuscript. All authors contributed to the article and approved the submitted version.

## Funding

This study received funding from Otago Innovation Ltd via RH. The funder was not involved in the study design, collection, analysis, interpretation of data, the writing of this article or the decision to submit it for publication. RM and LB are Q15 funded by MBIE Smart Idea Project UOOX1911. The ONE50 genome assembly is funded by Genomics Aotearoa project ‘High Quality Genomes and Population Genomics’ to AgResearch (JMEJ).

## Acknowledgments

We would like to thank Charles Hefer (AgResearch) for generating the draft genome assembly of ONE50 used in this study.

## Conflict of interest

The authors declare that the research was conducted in the absence of any commercial or financial relationships that could be construed as a potential conflict of interest.

## Publisher’s note

All claims expressed in this article are solely those of the authors and do not necessarily represent those of their affiliated organizations, or those of the publisher, the editors and the reviewers. Any product that may be evaluated in this article, or claim that may be made by its manufacturer, is not guaranteed or endorsed by the publisher.
