# Supplementary material for: Identification of the genes at S and Z reveals the molecular basis and evolution of grass self-incompatibility
Source: Front Plant Sci. 2022 Oct 18;13:1011299. doi: 10.3389/fpls.2022.1011299 (PMC9623065; doi:10.3389/fpls.2022.1011299)
Supplement: Supplementary file 1 [file Table_1.docx]

**Supplementary Table 1** – DUF247/SP/ZP annotations from other species used for expression analysis

| **Species/Gene** | **Gene ID** |
| --- | --- |
| *S.bicolor* |  |
| ZDUF_4 | SORBI_3006G239500 |
| ZSP | SORBI_3006G239600 |
| ZDUF_1 | SORBI_3006G239700 |
| SSP | SORBI_3010G144600 |
| SDUF_2 | SORBI_3010G144700 |
| *B.distachyon* |  |
| SSP | BRADI_2g35747v3 |
| SDUF_3 | BRADI_2g35750v3 |
| ZDUF_1 | BRADI_5g23930v3 |
| ZDUF_4 | BRADI_5g23932v3 |
| ZDUF_4 | BRADI_5g23934v3 |
| ZSP | BRADI_5g23936v3 |
| *T.aestivum* |  |
| SSP_A | TRIAE_CS42_1AS_TGACv1_019080_AA0060120.1 |
| ZSP_A4 | TRIAE_CS42_2AL_TGACv1_093422_AA0279940.1 |
| SSP_B | TRIAE_CS42_1BS_TGACv1_050274_AA0170030.1 |
| ZSP_D | TRIAE_CS42_2DL_TGACv1_158770_AA0525890.1 |
| ZSP_A1 | TRIAE_CS42_2AL_TGACv1_093422_AA0279950.1 |
| ZSP_B | TRIAE_CS42_2BL_TGACv1_131656_AA0430330.2 |
| SDUF_3A | TRIAE_CS42_1AS_TGACv1_020886_AA0080330.1 |
| SDUF_2B | TRIAE_CS42_1BS_TGACv1_049813_AA0161970.1 |
| ZDUF_4B | TRIAE_CS42_2BL_TGACv1_130467_AA0412090.1 |
| SDUF_2D | TRIAE_CS42_1DS_TGACv1_081396_AA0260520.3 |
| SDUF_3D | TRIAE_CS42_1DS_TGACv1_081196_AA0258610.1 |
| *O.sativa* |  |
| ZDUF_1 | LOC_Os04g55390.1 |
| ZDUF_4 | LOC_Os04g55370.1 |
| ZSP | LOC_Os04g55380.1 |
| SDUF_2 | LOC_Os05g10920.1 |
| SDUF_3 | LOC_Os05g10900.1 |
| SSP | LOC_Os05g10910.1 |
