# Supplementary material for: Identification of the genes at S and Z reveals the molecular basis and evolution of grass self-incompatibility
Source: Front Plant Sci. 2022 Oct 18;13:1011299. doi: 10.3389/fpls.2022.1011299 (PMC9623065; doi:10.3389/fpls.2022.1011299)
Supplement: Supplementary file 2 [file DataSheet_1.pdf]

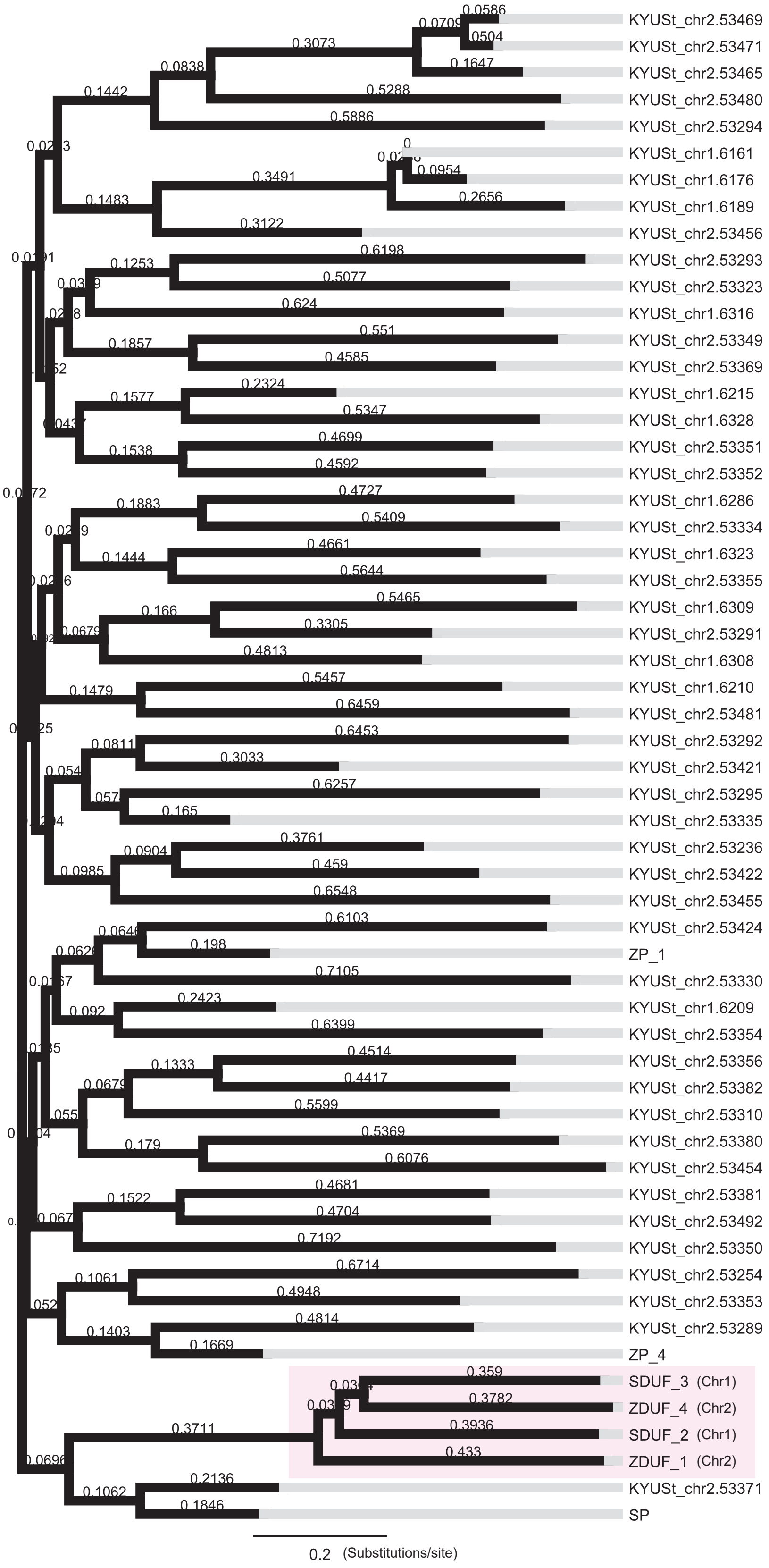

Supplementary Figure 1 Phylogenetic tree of all annotated proteins at the S- and Z-locus. A phylogenetic tree was constructed using proteins derived from both the S- (chromosome 1) and Z- (chromosome 2) loci. To produce the tree a global alignment with a BLOSUM62 cost matrix was performed, followed by tree building using a Jukes Cantor genetic distance model and nearest neighbor tree-building method. Only DUF247 proteins formed a significant clade containing genes from both loci/chromosomes (highlighted).

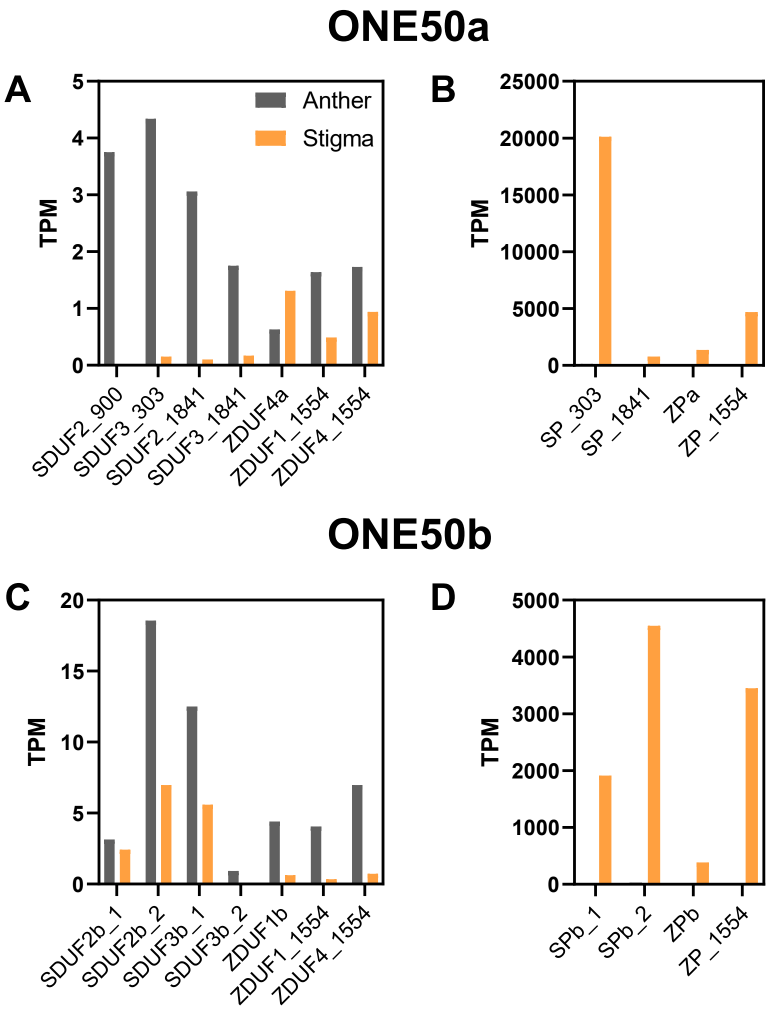

Supplementary Figure 2 Expression of assembled DUF/SP/ZP transcripts. Transcripts were assembled using Trinity software and expression was estimated using salmon. Trailing numbers indicate the presence of the transcript in the ONE50a genome and the relevant scaffold. (A) ONE50a DUF247 transcripts. (B) ONE50a SP/ZP transcripts (the ZPa transcript is concatenated with “ZDUF1a”). (C) ONE50b DUF247 transcripts. (D) ONE50b SP/ZP transcripts (ZPb is concatenated with “ZDUF4b”).

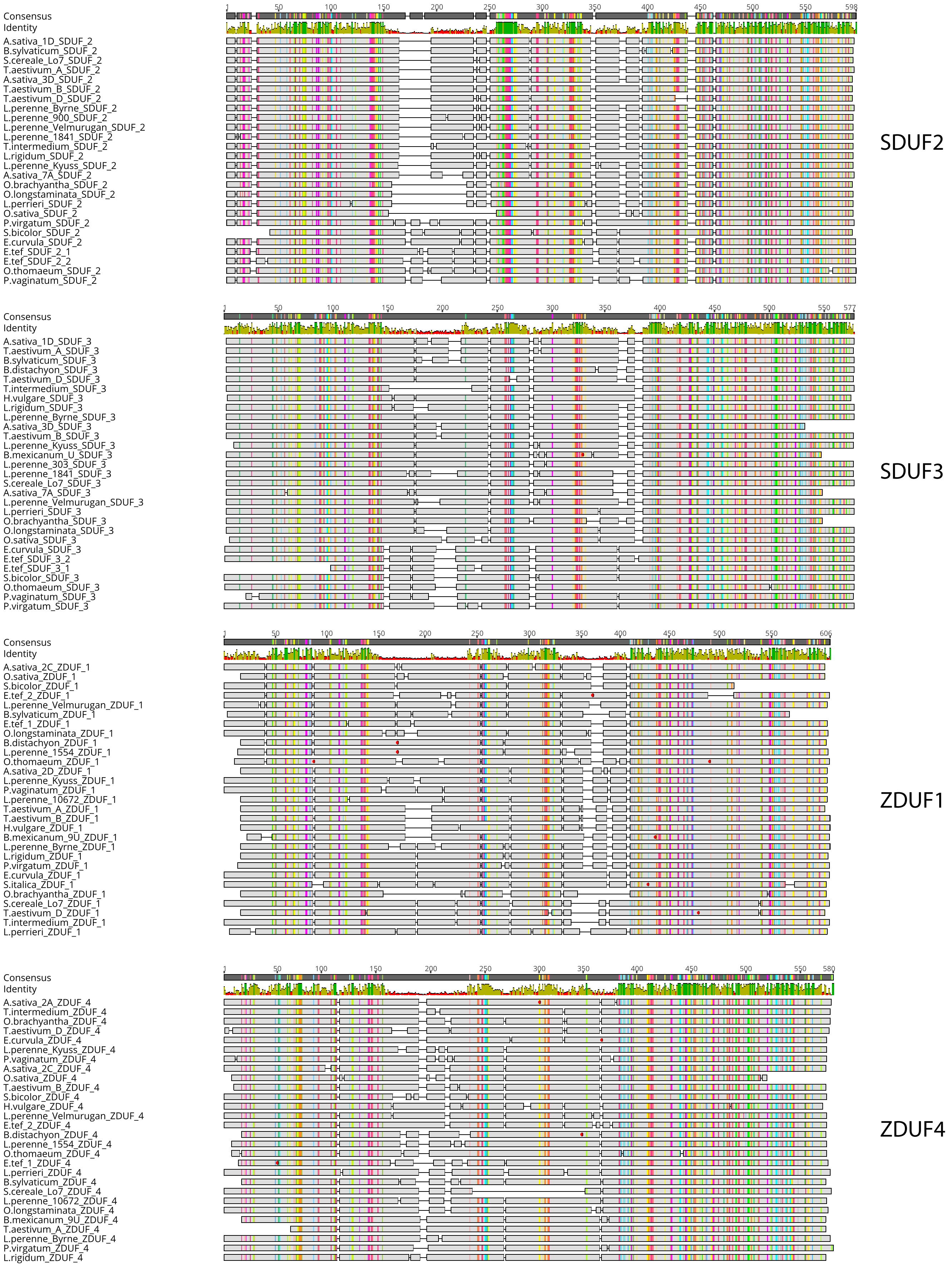

Supplementary Figure 3 Alignment of S- and Z-DUF247 proteins from grasses. Alignment using CLUSTALW with no free end gaps, a BLOSUM cost matrix, and gap open/extend penalty of 10/0.1. In cases where frameshift mutations or indels had occurred, sequences were reconstructed using tBLASTn or by translating and aligning all three frames of the relevant genomic sequence. Consensus and highlighting shows regions conserved in 90% of positions (very strict).

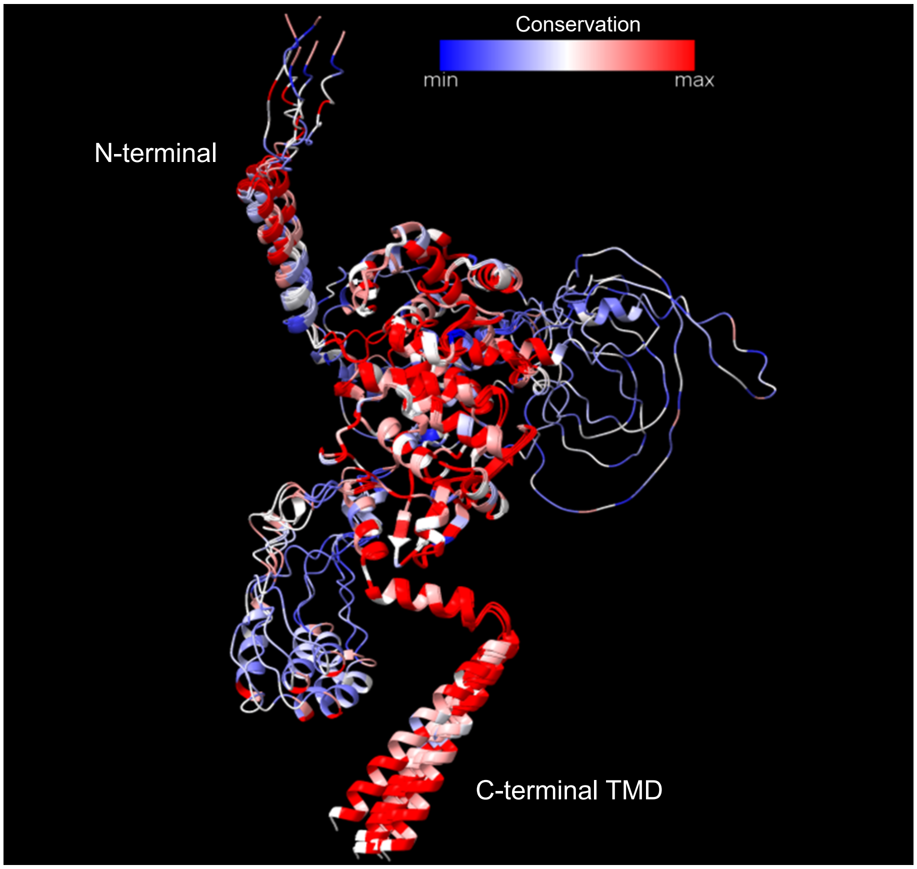

Supplementary Figure 4 AlphaFold Structure of SDUF2 from Ryegrass. AlphaFold was used to predict the structure of ~34 ryegrass SDUF2 proteins. A protein alignment was used to shade the structure from least homology (blue) to most homology (red).

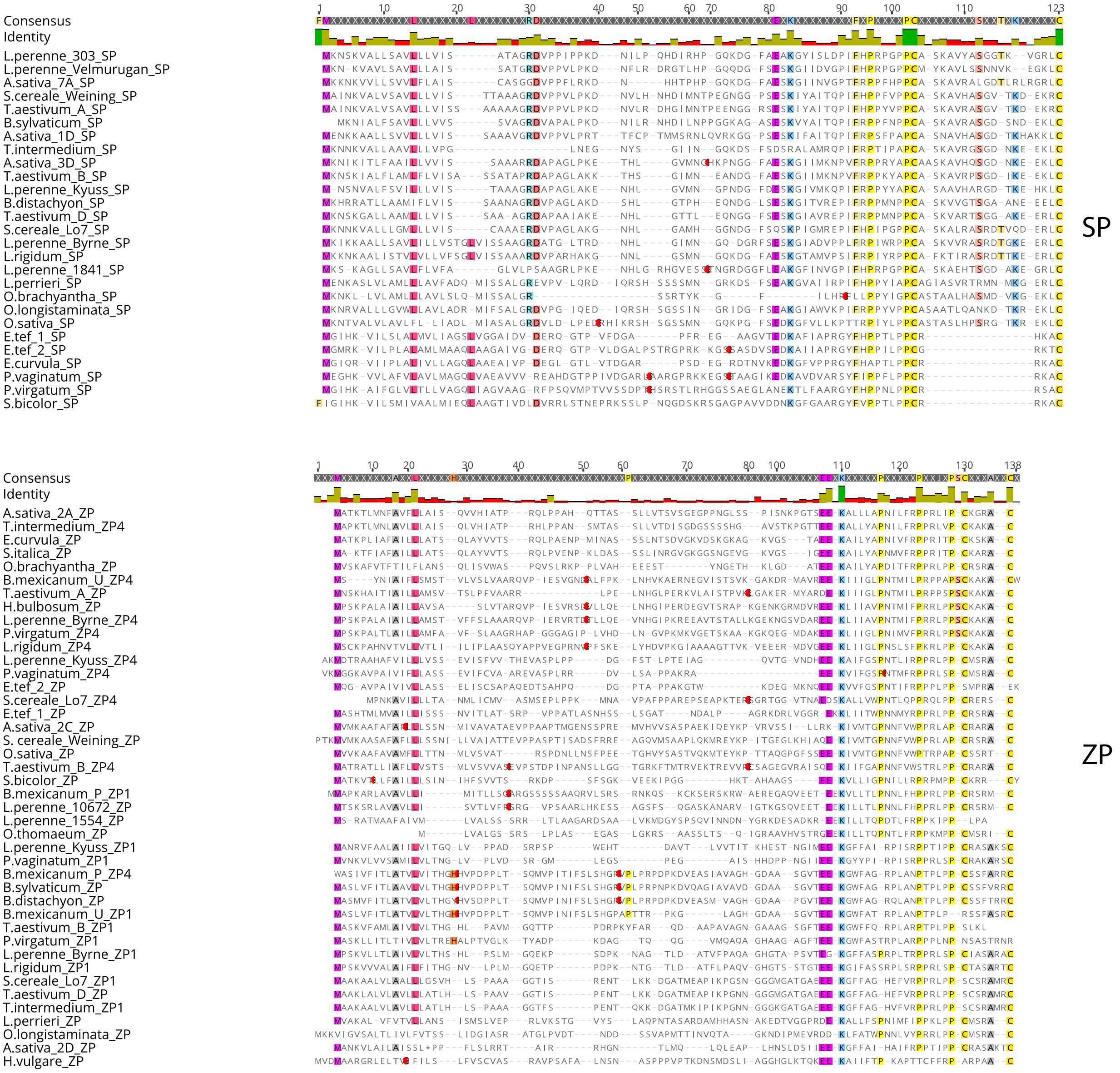

Supplementary Figure 5 Alignment of SP/ZP proteins from grasses. Alignment using CLUSTALW with no free end gaps, a BLOSUM cost matrix, and gap open/extend penalty of 10/0.1 Consensus and highlighting shows regions conserved in 75% of positions (strict).

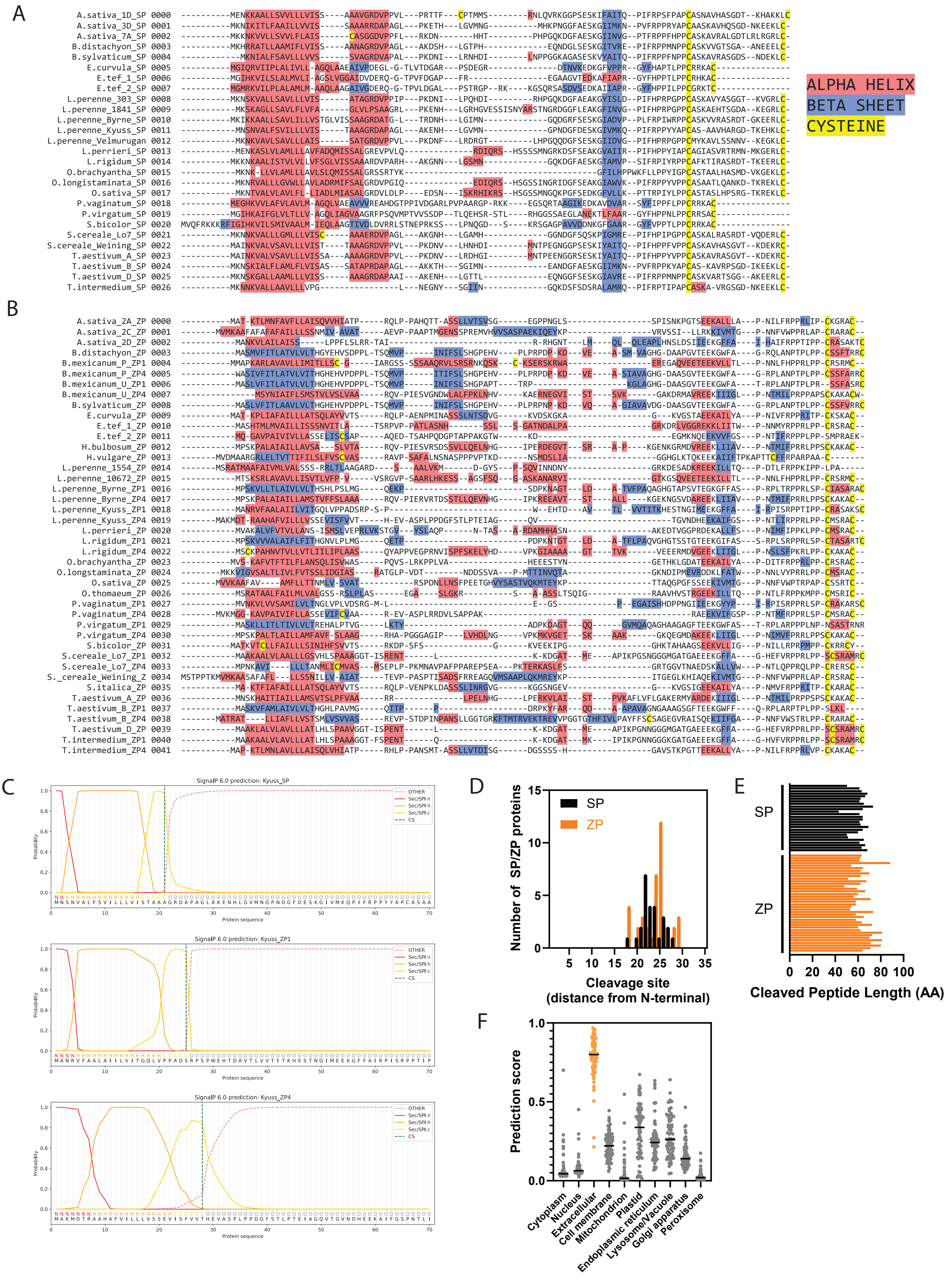

Supplementary Figure 6 Secondary structure and feature prediction of SP/ZP proteins. (A and B) Ali2d was used to detect alpha helices and beta sheets in SP (A) and ZP (B) proteins. Conserved Cysteines are also highlighted. (C) Exemplary output of SignalP6.0 on SP/ZP1/ZP4 from *L.perenne* (Kyuss). (D) Histogram showing predicted location of cleavage for all SP/ZP proteins examined. Five are omitted as SignalP6.0 did not predict cleavage. (E) Predicted length of cleaved peptide remaining (distance from cleavage site to C-terminal) for SP/ZP proteins. (F) Subcellular localization prediction using DeepLoc. Each SP/ZP is presented as a dot in each column with the relative probability predicted by DeepLoc. Most were predicted to be extracellular above all else.

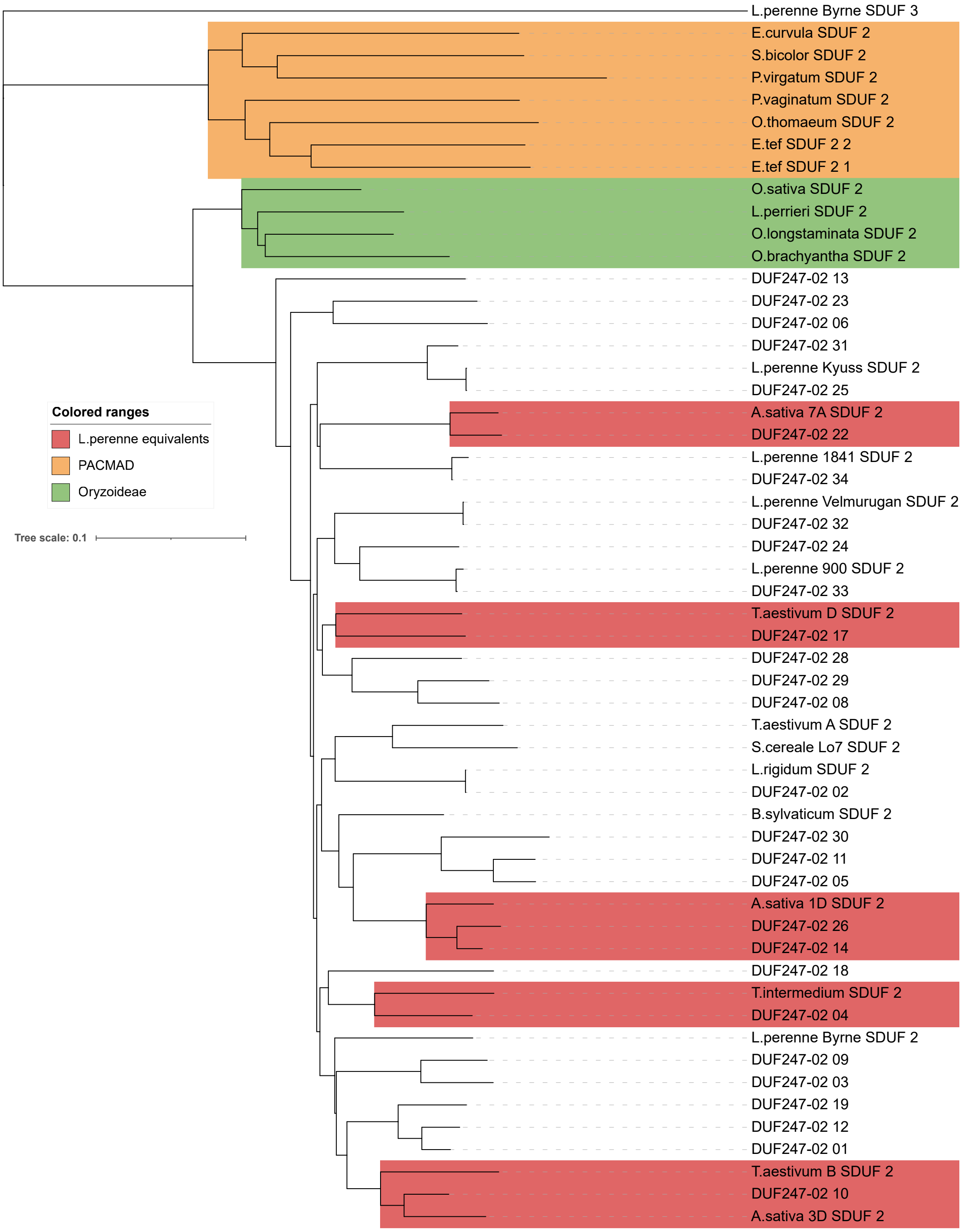

Supplementary Figure 7 Phylogenetic tree of SDUF2 proteins from *L.perenne* and other grasses. To produce the tree a global alignment with a BLOSUM62 cost matrix was performed, followed by tree building using a Jukes Cantor genetic distance model and nearest neighbor tree-building method. Highlighting indicates presence of a *L.perenne* equivalent of the identified Pooideae allele in the germplasm collection from Veeckman *et al.,* (2019). PACMAD and Oryzoideae do not have equivalent SDUF2 proteins in the ryegrass germplasm collection.

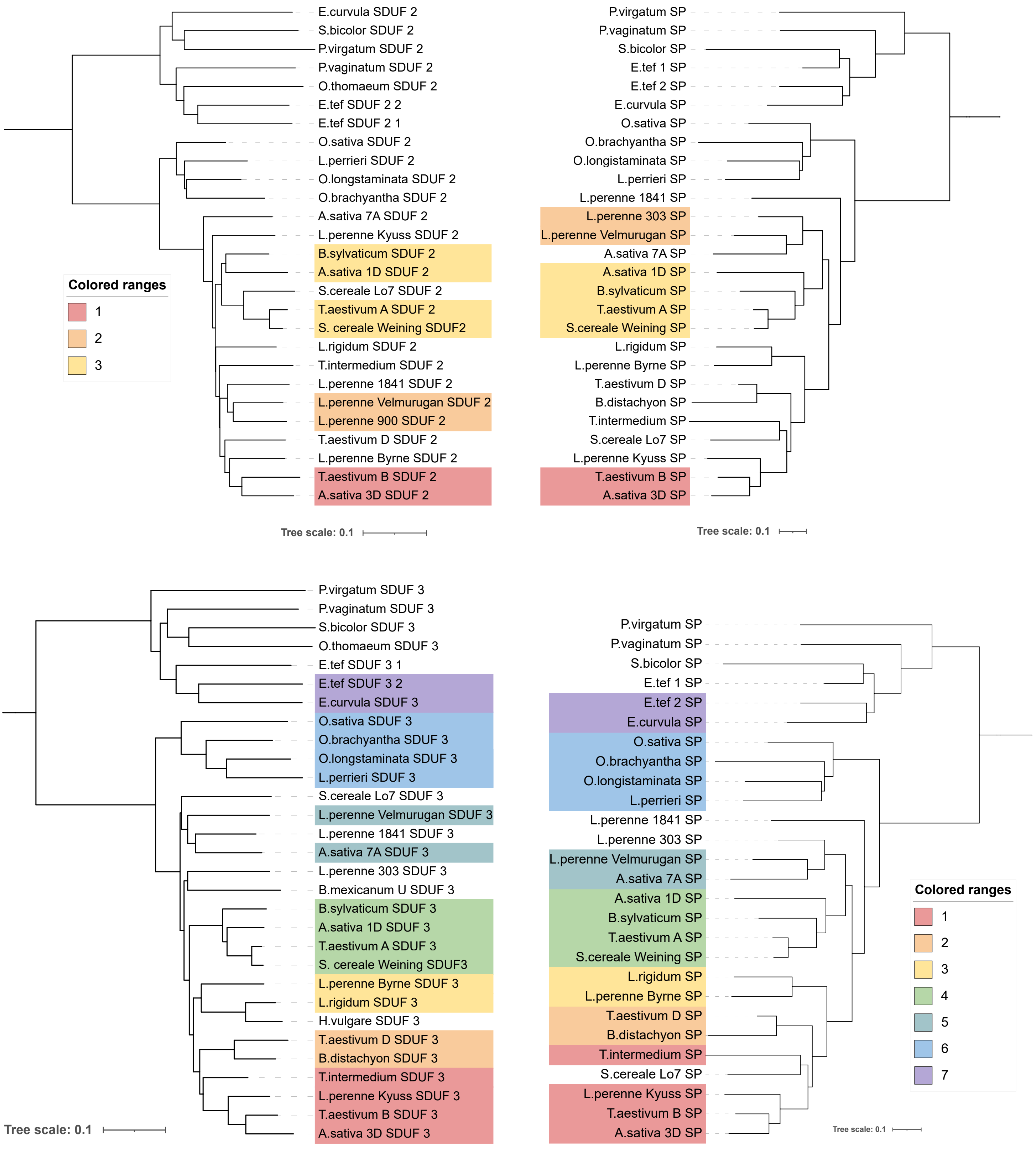

Supplementary Figure 8 Phylogenetic tree comparison of SDUF2/SDUF3 and SP. Pruned trees from Figure 5/6 are presented side by side. Highlights show clades conserved between adjacent trees, indicative of co-inheritance/co-evolution. Clade numbers are arbitrary.

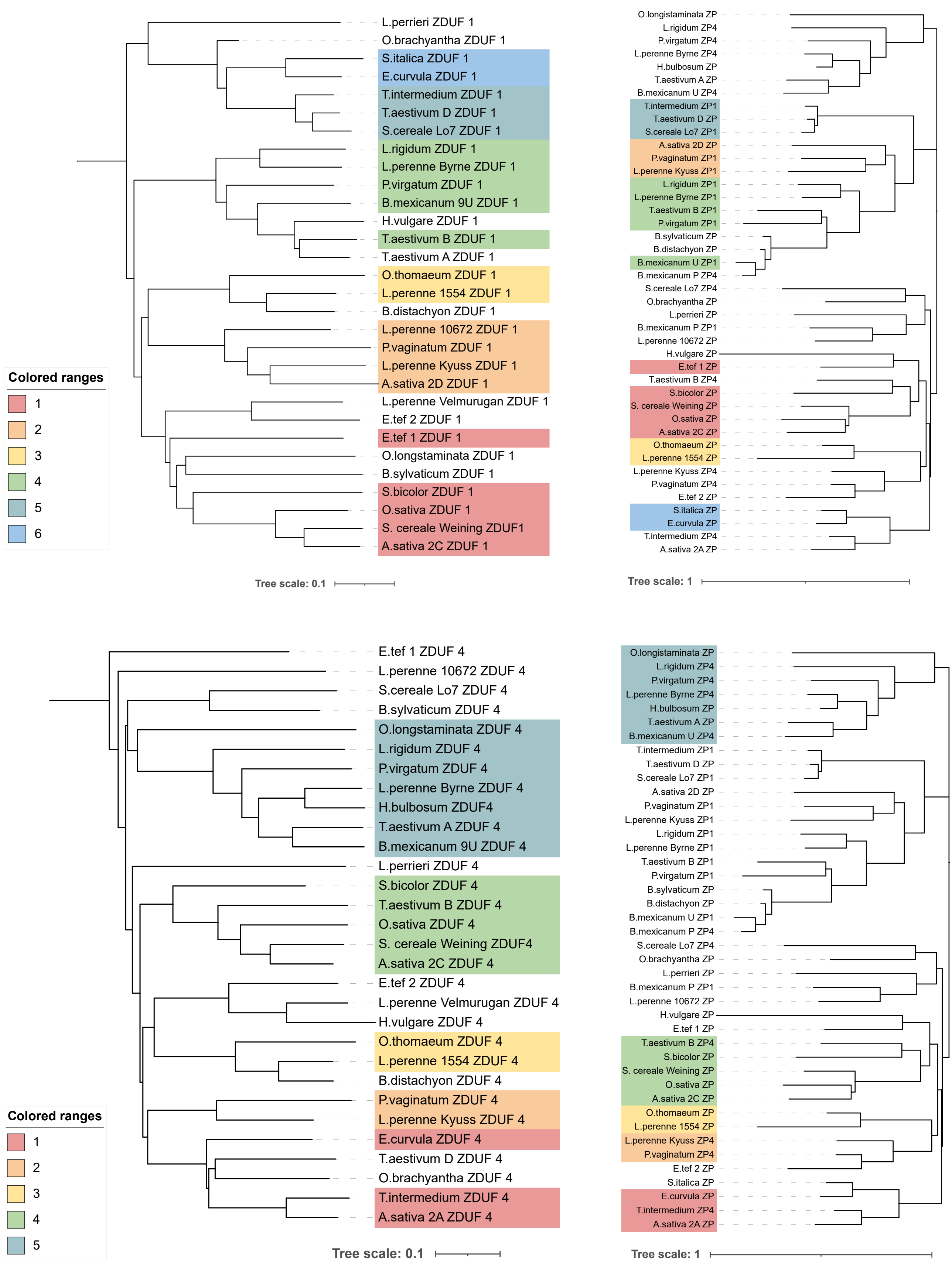

Supplementary Figure 9 Phylogenetic tree comparison of ZDUF1/ZDUF4 and ZP. Pruned trees from Figure 5/6 are presented side by side. Highlights show clades conserved between adjacent trees, indicative of co-inheritance/co-evolution. Clade numbers are arbitrary.
